# The Week's Work

**Published:** 1911-06-17

**Authors:** 


### The Week's Work.

### THE RENOVATION OF CHESTER INFIRMARY.

The latest report of the Chester Infirmary, which has just reached us, again alludes to our advocacy of the renovation scheme. The progress made up to the eve of this year's annual meeting was recorded in The Hospital of January 14 last. We now learn that "the extent to which the renovation can be carried is entirely a matter of finance," and it is hoped that the County Memorial to King Edward will remember the widespread public and municipal support which the extension scheme has received as the local memorial. The Pageant Committee, as we have already noted, has set a very good example, and we are very glad to learn that the Coronation will be used as a further opportunity of raising money for this very necessary purpose.

### SMALLPOX AND THE LONDON HOSPITAL.

The recent outbreak of smallpox in London did not cause anxiety only to the journalists and the staffs of the fever institutions. Last week's quarterly court revealed the fact that the London Hospital had the unpleasant, if not unnatural, experience of finding a case, admitted for quite other reasons, developing the disease. The unwitting admission of this incipient case was, like an unexpected fire-drill to another department, a severe practical test of the isolation methods in force. Fortunately these justified themselves, as no other cases occurred. We are glad to note the co-operation between the hospital and the public service in the person of Dr. D. L. Thomas, the Medical Officer of Health for Stepney, who has been recommended for addition to the list of life-governors for his help at this anxious moment.

### THE HORNSEY COTTAGE HOSPITAL MORTUARY.

The Hornsey Cottage Hospital, which was opened formally on January 27, 1910, already contemplates extension. We hear that "a properly equipped mortuary," a casualty ward, and an observation room are urgently needed. The need for such in every hospital was sufficiently emphasised in our Reports on the Hospitals of the United Kingdom to make it unnecessary to argue that question now. But it will surprise many to hear that a properly equipped mortuary was not included in the original plans.

### BOLTON INFIRMARY AND ROYAL RECOGNITION.

On June 3 we referred to a movement to gain royal recognition for the Bolton Infirmary, adding that "freedom from debt is, of course, one of the strongest facts in the favour of any hospital petitioning to be styled 'Royal.'" That fact, it now appears, has restrained many of the hospital's friends from supporting the movement, because, of the £48,000 appealed for as the result of Dr. Mackintosh's report, and to establish a memorial to the late King, only £25,000 has yet been subscribed. It seems to us, nevertheless, that the progressive movement which has been vigorous enough to secure £25,000 should not be allowed to stop there, but that the citizens of Bolton should be actively reminded that the aim of these efforts for a renovated infirmary is royal approval and royal recognition. That is the end that must be worked for, for it alone will show that the glaring needs and defects of the old institution have been remedied. The word "Royal" is not a magic adjective that secures additional income without additional effort. It is a recognition of work and persuasion that have been already done. We maintain most strongly that Bolton should work for it, and we believe that the inspiration that King Edward's name gave to the first half of the resuscitation of the Infirmary will not fail the hospital in completing the second half of its task in the name of King George. A further appeal for funds to complete the work and to enable a petition for the style of Royal to be presented with chance of success should be issued immediately. A public-spirited Mayor has here a great opportunuity.

### HOSPITAL FLAGS, THE RULE IN ENGLAND.

In the British Empire there is unfortunately no definite ruling on the point. Public hospitals—that is to say, hospitals which may definitely claim to be public institutions—and more particularly naval and military hospitals and a few royal hospitals (among them Bethlem) have the right to fly the national flag whenever they please. The Royal Standard cannot be flown by any building whether public or private unless by special sanction of the sovereign: it is the sovereign's private flag, as it were, and is only shown over a building in which the sovereign is resident or present at the time. Theoretically, a hospital may, with the sovereign's permission, fly the standard when the King or Queen is actually within the hospital precincts—as on the occasion of a royal visit of inspection; practically, however, the question of flying the standard never arises. The hospital flag so far as this country is concerned is the Union Jack; colonial hospitals will fly their own dominion or colonial ensigns, which consist essentially of the Jack with the arms or emblems of the dominion or colony upon it. While theoretically only public buildings can fly the national flag, it is now generally conceded that private individuals may also fly it by courtesy. A recent pronouncement on this point by Lord Crewe makes it clear that private individuals may fly the Union Jack over their houses or from staffs in their gardens. This is, however, merely a matter of courtesy: a voluntary hospital has no "right" to fly the Union Jack any more than the grocer or the tobacconist has. It may fly the municipal flag if it is associated with the borough in any corporate capacity, and it may also fly a special flag if it chooses to have one. In time of war when within the zone of operations it must fly the Red Cross flag in addition to the national ensign if it is a public hospital. Hospitals for foreigners, although owned by British subjects and on sites belonging to British subjects, may fly their own national emblems: as a matter of fact, the Jack is in such cases usually flown together with the foreign flag, equal prominence being given to the two ensigns. In the circumstances it seems clear that the proper flag for a hospital to fly is the Union Jack in the case of hospitals which may claim even remotely to be public institutions, and their own flag, together with the Jack, in the case of hospitals that are purely private institutions. When the Jack is flown care should be taken that it is properly flown: we have recently noticed many signals of distress over hospitals and public buildings both in London and in the provinces. Where a hospital is unsuccessfully appealing for public support, a reversed Union Jack may be an appropriate signal to fly: in other circumstances it is a sign of ignorance of a subject with which a tenderfoot scout is supposed to be thoroughly acquainted.

### THE INSPECTOR OF FACTORIES AND THE COUNTY HOSPITAL, CORK.

During the past year much building has been in progress at the South Charitable Infirmary and County Hospital, Cork. In the course of it his Majesty's Inspector of Factories arrived, and enjoined an increase of space in the laundry and the building of a new ironing-room. This visit is interesting, as it is only eighteen months ago since a test case decided that a hospital laundry is a factory within the meaning of the Acts of 1901 and 1907. The Hospital of November 13, 1909 (page 193), recorded the refusal of the Royal Berkshire Hospital, Reading, to admit the inspector, and the above legal decision, one effect of which is this recent laundry-building at Cork. The honorary architect to the county hospital there, Mr. James F. McMullen, was also busy supervising the new home for the outdoor staff of nurses, and the additions to the rooms for paying patients, including their ward-kitchen, admirable photographs of some of which are included in the current report. Indeed we must congratulate Mr. S. H. Newson, the hon. treasurer, or whomever is responsible, for making this document an illustrative as well as a statistical record of the work done.

### HOSPITAL DISPUTE AT COVENTRY.

The Coventry Division of the British Medical Association, on hearing that it was proposed to increase the out-patient staff at the Coventry and Warwickshire Hospital, has passed the following resolution: "That in the opinion of the Coventry Division of the British Medical Association, the existence of an out-patient department—except for the treatment of accidents, urgent cases, and those cases coming under the head of special departments, and also consultations—is not required in the public interest, and is calculated to lead to the squandering of money subscribed by the charitable public." Dr. Vaughan Pendred, the honorary secretary of the Division, in a covering letter to the local papers, states the grounds of this opinion to be the tendency of out-patient departments to simulate provident dispensaries, and the effect of the Insurance Bill, if carried, in increasing contract practice, and in thereby reducing the necessity of out-patient departments. Mr. Edward M. Iliffe, the vice-chairman of the hospital, thereupon has written to say that the committee has not appointed an almoner because it regards the abuse of this department as practically negligible, and that the hospital's honorary medical staff passed with practical unanimity a recommendation to increase its numbers owing to the great growth of out-patient work. This increase, Mr. Iliffe argues, will be justified even if the Insurance Bill passes, as the Bill covers only the wage-earner, and not his family. Mr. Iliffe then disputes the Association's contention that out-patient departments should be consultative in function on the ground that second opinions are only possible to people who can afford to pay for a first. This latter view, however, ignores the fact that hospitals, owing to the expense of many treatments, have a practical monopoly which no practitioner, whether in private or contract practice, can supply himself, and further, it assumes an antagonism between the practitioner and the hospitals, which exists only because out-patient departments have not been merely places of consultation or sorting centres in the past.

### THIS WEEK'S DRUG MARKET.

Practically no changes in value of any importance have taken place during the week, and business in the drug market has been extremely quiet, and will probably remain so until the end of the month. Carbolic acid shows a slight tendency to advance in price. Citric acid, tartaric acid, and cream of tartar are also likely to advance slightly if the hot weather continues. Cod-liver oil is a dull market, with a tendency to lower prices. Ipecacuanha tends dearer.

